# Diabetes Mellitus and COVID-19 in Adults: A Systematic Review of Pathophysiological Connections, Clinical Outcomes, and Therapeutic Considerations

**DOI:** 10.3390/ijms27083537

**Published:** 2026-04-15

**Authors:** Ioana-Madalina Mosteanu, Oana-Andreea Parliteanu, Beatrice Mahler, Adina Mitrea, Diana Clenciu, Adela Gabriela Stefan, Diana Cristina Protasiewicz Timofticiuc, Alexandru Stoichita, Mihaela Simona Popoviciu, Delia Viola Reurean Pintilei, Maria Magdalena Rosu, Theodora Claudia Radu Gheonea, Beatrice Elena Vladu, Lidia Boldeanu, Eugen Mota, Ion Cristian Efrem, Ionela Mihaela Vladu, Maria Mota

**Affiliations:** 1 Doctoral School, University of Medicine and Pharmacy of Craiova, 200349 Craiova, Romania; madalina.mosteanu@yahoo.com (I.-M.M.); eugenmota@yahoo.com (E.M.); mmota53@yahoo.com (M.M.); 2 Institute of Pneumophtisiology “Marius Nasta”, 050159 Bucharest, Romania; oana_andreea@yahoo.com (O.-A.P.); alexandru.stoichita@drd.umfcd.ro (A.S.); 3 Pneumology II Department, Faculty of Medicine, “Carol Davila” University of Medicine and Pharmacy, 050474 Bucharest, Romania; 4 Department of Diabetes, Nutrition and Metabolic Diseases, Faculty of Medicine, University of Medicine and Pharmacy of Craiova, 200349 Craiova, Romania; diana.clenciu@umfcv.ro (D.C.); theodora.gheonea@umfcv.ro (T.C.R.G.); ionela.vladu@umfcv.ro (I.M.V.); 5 Department of Diabetes, Nutrition and Metabolic Diseases, Calafat Municipal Hospital, 205200 Calafat, Romania; adela.firanescu@yahoo.com; 6 Department of Diabetes, Nutrition and Metabolic Diseases, Faculty of Midwives and Nursing, University of Medicine and Pharmacy of Craiova, 200349 Craiova, Romania; diana.protasiewicz@umfcv.ro (D.C.P.T.); maria.rosu@umfcv.ro (M.M.R.); 7 Faculty of Medicine and Pharmacy, University of Oradea, 410073 Oradea, Romania; mihaela.popoviciu@didactic.uoradea.ro; 8 Department of Medical-Surgical and Complementary Sciences, Faculty of Medicine and Biological Sciences, “Stefan cel Mare” University, 720229 Suceava, Romania; delia.pintilei@usm.ro; 9 Department of Diabetes, Nutrition and Metabolic Diseases, Consultmed Medical Centre, 700544 Iasi, Romania; 10 Faculty of Medicine, University of Medicine and Pharmacy of Craiova, 200349 Craiova, Romania; beatricevladu75@gmail.com; 11 Department of Microbiology, Faculty of Medicine, University of Medicine and Pharmacy of Craiova, 200349 Craiova, Romania; lidia.boldeanu@umfcv.ro; 12 Department of Internal Medicine—Medical Semiology, Faculty of Dentistry, University of Medicine and Pharmacy of Craiova, 200349 Craiova, Romania; cristian.efrem@umfcv.ro

**Keywords:** diabetes mellitus, COVID-19, SARS-CoV-2, hyperglycemia, antidiabetic therapy, PRISMA

## Abstract

The disproportionately severe disease course of diabetic patients with SARS-CoV-2 infection was repeatedly observed by clinicians during the COVID-19 pandemic. The overlap between metabolic impairment, viral pathophysiology, and chronic inflammation created a pattern that urged deeper examination. The aim of this paper was to review and synthesize evidence regarding the interaction between diabetes mellitus and COVID-19. We synthesized evidence across mechanistic pathways (immune dysregulation, chronic inflammation, ACE2/DPP-4-related signaling, endothelial dysfunction, and pancreatic involvement) and key clinical outcomes (severity, intensive care unit (ICU) admission, mortality, dysglycaemia/new-onset diabetes, and DKA). This systematic search was conducted in PubMed, Clinical Key, and Google Scholar. The eligibility criteria included papers on adults (≥18 years) with pre-existing diabetes mellitus (type 1 or type 2) or newly diagnosed diabetes/hyperglycemia and confirmed SARS-CoV-2 infection, published between January 2020 and October 2025, in English language. The PRISMA guidelines were used for data extraction. We identified 412 articles, out of which only 30 met all the inclusion criteria. Diabetes was consistently evoked as a major risk factor for severe COVID-19, being associated with higher susceptibility to pneumonia, respiratory failure, ICU admission, and mortality. The explanation lies in the impaired immune system, endothelial dysfunction, and metabolic repercussions imposed by hyperglycemia. Several antidiabetic drugs appeared protective in multiple cohorts. In conclusion, the accumulated evidence underscores the tight interplay between metabolic disease and COVID-19. Essentially, the clinical management of these patients would be a thoughtful selection of antidiabetic therapy and close metabolic monitoring.

## 1. Introduction

Diabetes mellitus appeared to be the most common comorbidity identified among patients with severe acute respiratory syndrome coronavirus-2 (SARS-CoV-2) infection [1]. Compared to the population without diabetes, patients with diabetes seemed more probable to require oxygen supplementation, present clinical deterioration, and to develop acute respiratory distress that required admission to the intensive care unit (ICU) [2]. These clinical observations were soon sustained by literature data showing a strong relationship between diabetes and the increased risk of coronavirus disease-19 (COVID-19) severity. Clinical reports noted that the majority of deceased patients (65%) had pre-existing conditions, most frequently represented by diabetes, hypertension, heart failure, chronic kidney disease, and obesity [3], underscoring the cumulative metabolic and cardiovascular burden present in this study sample.

The mechanisms underlying this association are multifactorial and extend beyond hyperglycemia alone. Diabetes is associated with immune dysregulation, impaired host defense, and a chronic pro-inflammatory state, which may contribute to an exaggerated response to viral infections [4]. Hypertension, obesity, and endothelial dysfunction are seen as risk factors in many individuals with diabetes, and these contribute to the vulnerability during acute respiratory distress.

Considerable attention was drawn by angiotensin-converting enzyme-2 (ACE2) and dipeptidyl peptidase-4 (DPP-4) [5] receptors, playing a role in the glucose homeostasis, but also in viral pathophysiology. Their expression can be influenced by hyperglycemia and by certain antidiabetic therapies, which may partly explain the heterogeneity of outcomes among treated patients. SARS-CoV-2 seems to be capable of influencing glucose levels, either through systemic inflammation or direct pancreatic involvement, raising the possibility of new-onset diabetes following infection [6,7].

This review aims to analyze research published between 2020 and 2025 to clarify how diabetes influences COVID-19 progression, to explore mechanism links, and to summarize the impact of commonly used antidiabetic medications.

## 2. Materials and Methods

This systematic review was conducted using the Preferred Reporting Items for Systematic Reviews and Meta-Analyses (PRISMA) 2020 guidelines [8], and the completed PRISMA checklist is provided in the Supplementary Materials. The literature search was performed using PubMed, Google Scholar, and Clinical Key, from papers published between 1 January 2020 to 30 October 2025. The following words were used in combination: “diabetes mellitus” OR “hyperglycemia” OR “diabetes” AND “COVID-19” OR “SARS-CoV-2”, AND “adults” AND “diabetic ketoacidosis”, AND “antidiabetic therapy”, AND “clinical outcomes” AND “2020/01/01”: “2025/10/30”. Additional references were identified from the bibliography of relevant papers. The meeting criteria for inclusions were: studies including adult patients (≥18 years) with pre-existing or newly diagnosed diabetes and confirmed SARS-CoV-2 infection; reported clinical outcomes, mechanistic data, or therapeutic implications; original research articles (cohorts, case–control, cross-sectional, trials), case series or systematic reviews; English must have been the language used in the research.

The exclusion criteria were: non-clinical or laboratory-only studies without direct relevance to diabetes; editorials, commentaries, and conference abstracts without extractable data; pediatric populations; studies lack information specific to diabetic patients.

Study selection was made based on titles and abstracts that were screened independently by two reviewers. Full texts were examined when eligibility was unclear. When in doubt about a paper, a third more experienced author made the final decision regarding the paper. Data extraction included: authors, year of publication, study design and country, sample size, characteristics of the diabetic cohort, outcomes related to severity and mortality, observations on glucose metabolism, and the effect of antidiabetic therapies, when reported.

**Figure 1:**
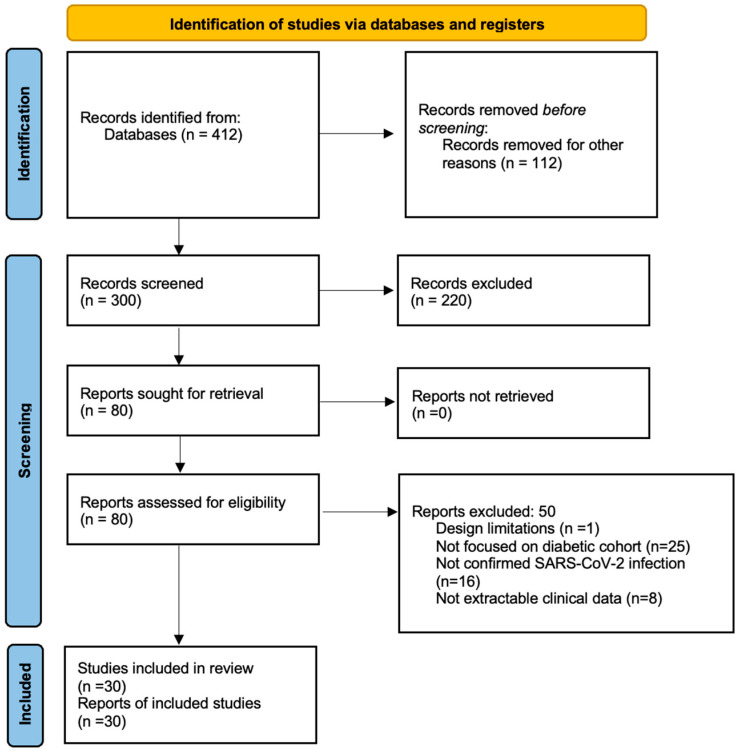
PRISMA Flow-chart summarizing the study selection process.

The initial search produced 412 records. After the removal of duplicates, 300 titles and abstracts underwent screening. Of these, 80 research articles were reviewed in detail, and a total of 30 studies met the inclusion criteria and were incorporated into the final synthesis. The study selection process is summarized in the PRISMA flow diagram (Figure 1) [8].

For each included study, we extracted the following variables: authors, year of publication, country, study design, cohort characteristics, diabetes type when reported, sample size or study scope, outcomes evaluated, and main conclusions relevant to the interaction between diabetes and COVID-19. In addition to original clinical studies, high-quality narrative, mechanistic reviews, and therapeutic implications were included to contextualize clinical findings.

Risk-of-bias assessment was conducted using validated tools tailored to each study design. Observational studies were evaluated using the Newcastle–Ottawa Scale (NOS), case series were assessed with the Joanna Briggs Institute (JBI) critical appraisal checklist, and systematic reviews or meta-analyses were appraised using the Risk of Bias in Systematic Reviews (ROBIS) tool.

The PROSPERO registration was not applicable, as this research does not involve therapeutic intervention on human subjects. Therefore, our paper was registered in the OSF Registries.

## 3. Results

Included studies are summarized in Table 1 and grouped by study design (clinical studies, case series, systematic reviews/meta-analyses, and narrative/mechanistic reviews), with each group ordered chronologically.

### 3.1. Characteristics of Included Studies

As detailed in Table 1, the body of evidence included 30 studies, spanning a range of study designs: 9 clinical studies, 1 case series, 4 meta-analyses, and 16 narrative and mechanistic reviews. These investigations were conducted in varied clinical and research settings across Europe, Asia, and North America, ensuring broad geographic representation of the available literature.

Across included studies, the most consistently reported clinical outcomes were higher rates of severe disease, ICU admission, and mortality among adults with diabetes, particularly when hyperglycaemia was present at admission or during hospitalization. Mechanistic reviews converged on immune-metabolic dysregulation, endothelial injury, and altered receptor pathways (ACE2/DPP-4) as plausible contributors to worse pulmonary and systemic outcomes.

### 3.2. Clinical Outcomes in Diabetic vs. Non-Diabetic Patients

The findings were consistent across different study designs and populations, reinforcing the association between diabetes and increased risk of severe COVID-19 outcomes.

In the CORONADO study by Cariou et al. [10], which included 1317 inpatients with diabetes, with a predominance of male patients (64%), almost 30% required invasive or non-invasive ventilatory support, and early mortality was high, confirming that pre-existing diabetes is closely linked to severe pneumonia and death. These findings are supported by the prospective Romanian cohort of Parliteanu et al. [17], in which 813 hospitalized patients with pulmonary impairment were analyzed, resulting that admission hyperglycemia, regardless of prior diabetes status, correlated with more severe respiratory distress and poorer radiological evolution. Large population-based analyses [29] demonstrated a significantly increased risk of severe COVID-19 outcomes and mortality in patients with diabetes compared with non-diabetic patients.

The multicenter cohort of Lalau et al. [13] further underlined the prognostic impact of glycemic status: among 2449 hospitalized patients with diabetes and COVID-19, those with better baseline glycemic control and ongoing metformin therapy had lower in-hospital mortality. In parallel, narrative and clinical reviews [9,11,14,20,24] repeatedly reported higher rates of ICU admission, need for mechanical ventilation, and death among diabetic versus non-diabetic patients across different health systems.

Importantly, the systematic review and meta-analysis by Shrestha et al. [19] showed that new-onset diabetes during COVID-19 is also associated with significantly higher risk of severe disease and mortality compared with non-diabetic individuals. Together with the meta-analysis by Zhou et al. [22], which demonstrated an increased long-term incidence of diabetes after COVID-19 infection, these data suggest that both pre-existing and newly developed disturbances in glucose homeostasis identify patients at particular risk of adverse outcomes.

### 3.3. Mechanistic Links: Viral Entry, Inflammation, Metabolic Dysfunctions

SARS-CoV-2 infection may influence glucose metabolism through several interconnected mechanisms involving pancreatic injury, systemic inflammation, and metabolic dysregulation. The presence of ACE2 receptors in pancreatic islets suggests that SARS-CoV-2 may directly affect β-cell function and insulin secretion, potentially contributing to acute hyperglycemia and the development of new-onset diabetes during infection [23,25].

Severe infection is characterized by the release of pro-inflammatory cytokines such as interleukin-6 (IL-6), tumor necrosis factor-α (TNF-α), and other mediators that can induce insulin resistance and worsen glycemic control in patients with pre-existing diabetes [29,30]. This inflammatory state may further impair endothelial function and promote a pro-thrombotic environment, contributing to the increased risk of complications observed in diabetic patients with COVID-19 [27].

Clinical evidence also suggests that disturbances in glucose metabolism may emerge during SARS-CoV-2 infection. Several studies have reported cases of newly diagnosed hyperglycemia or diabetes in patients hospitalized with COVID-19, possibly reflecting both stress-induced metabolic alterations and direct pancreatic involvement [18,19]. These metabolic abnormalities have been associated with poorer clinical outcomes, including increased disease severity and mortality [10].

Together, these findings indicate that the interaction between SARS-CoV-2 infection and diabetes is complex and multifactorial, involving systemic inflammation, metabolic dysregulation, and impaired glucose homeostasis, which collectively contribute to increased disease severity [23,29].

### 3.4. ACE2 Dysregulation and Endothelial Dysfunction

Beyond its role in viral entry, ACE2 dysregulation contributes to endothelial dysfunction and vascular injury in COVID-19, which are particularly relevant in patients with diabetes. In these patients, chronic hyperglycemia, insulin resistance, and associated comorbidities contribute to alterations in ACE2 expression across multiple tissues, including the lung, heart, kidney, and adipose tissue [23,34]. This imbalance in the renin–angiotensin–aldosterone system promotes vasoconstriction, endothelial damage, and prothrombotic state. This helps explain why diabetic patients more frequently develop diffuse alveolar damage, microthrombosis, and multi-organ involvement.

### 3.5. DPP-4, Immune Modulation and Metabolic Signalling

The reviews by Bassendine et al. [27] and Drucker [23] highlight the dual role of DPP-4. Beyond cleaving incretin hormones, DPP-4 acts as a co-stimulatory molecule on immune cells and has been exposed as a potential co-receptor for coronaviruses. In diabetes, DPP-4 expression and activity are often increased. This may amplify pro-inflammatory signaling and T-cell activation, contributing to the exaggerated cytokine response observed in severe COVID-19. At the same time, interference with the incretin system affects insulin secretion and glucagon suppression, favoring hyperglycemia and further perpetuating immune-metabolic dysregulation.

### 3.6. Hyperglycemia and Immune Dysfunction

High glucose levels reduce neutrophil chemotaxis and phagocytosis, alter complement function and promote non-enzymatic glycation of immunoglobulins. On the adaptive side, T-cell responses are delayed or blunted, while the baseline secretion of pro-inflammatory cytokines (IL-6, TNF-α, IL-1β) is increased. When SARS-CoV-2 infection occurs on this background, host reaction is both less efficient in clearing the virus and more prone to cytokine storm, leading to extensive lung and systemic injury, all these explaining the viral clearance noted by [14].

### 3.7. Metabolic and Viral Inflammation

The interaction between metabolic dysfunction and inflammation plays a central role in the severity of COVID-19 in patients with diabetes. Chronic hyperglycemia is associated with increased oxidative stress, immune dysregulation, and activation of pro-inflammatory pathways, which may exacerbate the host response to SARS-CoV-2 infection.

Importantly, T1DM and T2DM differ in their underlying pathophysiology. T1DM is an autoimmune disease associated with oxidative stress and an increased risk of DKA, particularly during acute infections such as COVID-19 [39,40,41]. In contrast, T2DM is characterized by insulin resistance and chronic low-grade inflammation, often linked to obesity and adipose tissue dysfunction [42].

In obesity, adipose tissue is characterized by increased infiltration of pro-inflammatory macrophages and enhanced secretion of adipokines and cytokines, which maintain a state of chronic low-grade inflammation. When SARS-CoV-2 infection superimposes, cytokine production is amplified, endothelial activation is enhanced and pro-thrombotic pathways are triggered. This synergy between metabolic inflammation and viral inflammation provides a plausible explanation for the disproportionate severity of COVID-19 in obese diabetic patients [43].

### 3.8. Pancreatic Involvement and β-Cell Dysfunction

The case series of Suwanwongse and Shabarek [18] and multiple reviews [30,31,34,40] suggest that SARS-CoV-2 can directly or indirectly affect pancreatic β-cell function. ACE-2 expression that has been detected in pancreatic islets, raising the possibility of direct viral cytotoxicity. In addition, systematic inflammation, hypoxia, corticosteroid treatment and catecholamine surges all induce insulin resistance and β-cell stress. The result can be acute decompensation of previously compensated diabetes or true new-onset diabetes, often presenting with DKA, as described in the case series [16]. These mechanisms are consistent with the higher prevalence and poor prognosis of new-onset diabetes reported in the meta-analyses [19,22].

### 3.9. Drug-Specific Clinical Outcomes

The effects of antidiabetic therapies on COVID-19 outcomes have been summarized in Table 2.

Metformin significantly reduced mortality in multiple cohorts when used, due to its beneficial effects through AMP-protein kinase (AMPK) activation, endothelial protection, and anti-inflammatory actions, potentially mitigating the dysregulated inflammatory and vascular pathways observed in severe COVID-19. These mechanisms fit well with the clinical findings from Ganesh and Randall [20] and Lalau et al. [13] where metformin use was consistently associated with lower mortality among hospitalized COVID-19 patients with diabetes.

From a clinical perspective, observational studies evaluating DPP-4 inhibitors in patients with COVID-19 and diabetes have yielded inconsistent results. Most population-level observational studies reported neutral effects on clinical outcomes; however, one Italian retrospective study reported reduced mortality among patients treated with sitagliptin [11], whereas larger analyses from Italy and China did not demonstrate significant improvements in major endpoints [9,14]. Mechanistically, DPP-4 inhibition has been hypothesized to modulate immune signaling and inflammatory responses during SARS-CoV-2 infection [27]. Nonetheless, the available evidence remains inconclusive, and any potential benefit may be context-dependent, given the predominantly observational nature of current data [21,35].

SGLT2-i therapies and GLP-1RA were observed to reduce macrophage-driven inflammation and were associated with fewer ICU admissions and reduced mortality supported by findings in Monda et al. [16] and Chen et al. [21] studies. The large European database study by Foresta et al. [35] confirmed that GLP-1RA and SGLT2i users experienced improved outcomes. These drugs likely confer benefit by combining good glycemic control with weight reduction, cardiorenal protection and anti-inflammatory effects, thereby attenuating several of the mechanistic pathways that make diabetic patients vulnerable.

The role of thiazolidinediones, particularly pioglitazone, remains largely theoretical. Although their anti-inflammatory activity and potential to modulate ACE2 expression have been described [44,45], and some in silico analyses suggest antiviral effects [46], the known risks of fluid retention and heart failure limit their attractiveness during acute respiratory illness [47,48,49]. Future studies may clarify whether targeted subgroups could benefit, but current evidence does not support routine use for COVID-19.

Overall, available evidence suggests that some antidiabetic therapies may influence COVID-19 outcomes through metabolic and anti-inflammatory mechanisms, although most current data are derived from observational studies.

### 3.10. New-Onset Diabetes After COVID-19

Several studies have reported the occurrence of newly diagnosed diabetes during or after SARS-CoV-2 infection. In some patients, hyperglycemia or DKA has been the initial manifestation, suggesting that COVID-19 may precipitate metabolic decompensation in susceptible individuals [18]. The development of new-onset diabetes in this context is likely multifactorial and may involve direct β-cell dysfunction, systemic inflammatory responses leading to insulin resistance, and the effects of glucocorticoid therapy frequently used in severe COVID-19 cases [30].

Evidence from systematic reviews and meta-analyses further supports the association between SARS-CoV-2 infection and disturbances in glucose metabolism. For example, a meta-analysis reported that newly diagnosed diabetes and stress hyperglycemia were associated with increased disease severity and higher mortality compared with normoglycemic patients [19]. Similarly, recent analyses suggest that SARS-CoV-2 infection may increase the risk of incident diabetes during the post-acute phase of the disease [22].

Clinical observations have demonstrated alterations in glucose homeostasis among hospitalized patients with COVID-19, including worsening glycemic control and the emergence of previously undiagnosed diabetes [17]. These findings emphasize the importance of long-term metabolic surveillance following SARS-CoV-2 infection, particularly in individuals with pre-existing metabolic risk factors [31].

## 4. Discussion

A unifying theme across the studies reviewed is the impairment of the immune response in individuals with diabetes. Several authors describe delayed or blunt adaptive immunity in the presence of chronic hyperglycemia, particularly in patients with T2DM, where comorbidities such hypertension, obesity, cardiovascular disease, and advanced age frequently coexist. The mechanisms are multifactorial: hyperglycemia interferes with leukocyte function, disrupts cytokine signaling, and increases baseline inflammatory tone, creating a physiological cascade. These pathophysiological pathways were highlighted early in mechanistic studies examining ACE2 and DPP-4 biology, which together provide a biological rationale for the heightened vulnerability of diabetic patients.

One consistent observation across clinical cohorts is the strong association between hyperglycemia at admission and respiratory distress. This was confirmed in analyses from large multinational cohorts to local hospital studies, including a recent report highlighting the correlation between peak glucose levels and severity of pulmonary impairment [17,19]. These findings reinforce the idea that acute dysglycaemia is not merely a marker of severity, but an active participant in disease progression.

A second major conclusion emerging from the reviewed literature is the possibility of diabetes developing after SARS-CoV-2 infections. Case reports and cohort studies described new-onset diabetes, often accompanied by DKA, both at presentation and weeks to months after apparent recovery [18,50]. Several mechanisms have been proposed: direct pancreatic injury, cytokine-mediated beta-cell dysfunction, unmasking of previously undiagnosed disease, and the metabolic effects of corticosteroids used in treatment protocols [30,51]. Although the true prevalence remains uncertain, the pattern is consistent enough to warrant long-term monitoring of glucose metabolism in post-COVID patients.

Post-COVID-19 syndrome involves multiple organ systems. Emerging evidence suggests that SARS-CoV-2 infection may also affect skeletal health, with studies reporting a decline in bone mineral density associated with disease severity and clinical outcomes [37,52]. This may be particularly relevant in patients with diabetes, who are already at increased risk of altered bone metabolism and fragility.

From a therapeutic standpoint, antidiabetic medications have been examined extensively for their influence on COVID-19 outcomes. Among these, metformin stands out as the drug with the most consistently favorable signals. Its anti-inflammatory, endothelial-stabilizing, and AMP-activated protein kinase (AMPK) actions have been well described in molecular and translational studies [32,36,53,54]. Several large analyses reported reduced mortality among metformin users [13,20], and a systematic review examining its role across diverse populations supported these findings [20]. While caution remains necessary in patients with hypoxia or hemodynamic instability due to the risk of lactic acidosis, the overall evidence suggests that continuing metformin in stable patients is beneficial.

The evidence regarding DPP-4i is more heterogeneous. While some experimental papers describe DPP-4 as a potential viral co-receptor [55], large population-level studies from Italy and France did not find significant improvements in clinical outcomes among users [10,14,28,56]. Conversely, a multicenter Italian study suggested that sitagliptin at hospitalization may reduce mortality and improve clinical status [11]. Other cohorts exported neutral or even unfavorable associations [9,12]. The mixed nature of these findings indicates that DPP-4i likely do not exert uniform effects across patient groups and should not be initiated specifically for COVID-19 management, although continuation in stable patients appears safe.

SGLT2 inhibitors and GLP-1RA represent an evolving area of interest. Recent mechanistic and clinical data suggest that SLGT2i may reduce inflammatory signaling, improve endothelial function, and potentially lower the risk of respiratory deterioration [38,57,58,59]. Observational studies from Italy report fewer ICU admissions among users [16], while a Bayesian network meta-analysis noted a lower mortality rate associated with this drug class [21]. GLP-1RA, meanwhile, offers stable glycemic control with minimal hypoglycemia, which may be advantageous during acute illness. Their therapeutic profile and early observational data suggest a possible reduction in adverse outcomes [35,60,61]. As with SLGT2i, however, more targeted trials are needed before definitive recommendations can be made.

The interaction between diabetes and COVID-19 extends beyond the acute phase. Post-COVID syndrome, a source of ongoing clinical concern, often manifests with persistent fatigue, respiratory symptoms, or metabolic instability. Several authors have documented this trajectory in both general populations and vulnerable groups, including individuals with diabetes [22,31,62]. The persistence of hyperglycemia after acute infection has important implications for long-term care and supports the need for structured follow-up.

Lifestyle disruptions during the pandemic also contributed to worse glycemic control, as noted in retrospective analyses from Asia and Europe [15]. These findings remind clinicians that the broader context of a health crisis can indirectly affect metabolic stability, even in patients who have not contracted the virus.

Taken together, the evidence paints a cohesive picture: diabetes predisposes patients to more severe COVID-19, through intertwined metabolic, immunologic, and vascular pathways [63]. Acute hyperglycemia is consistently associated with poorer outcomes regardless of prior diagnosis. SARS-CoV-2 infection can precipitate or unmask diabetes. Metformin appears protective in stable patients, while SGLT2 inhibitors and GLP-1RA show promising but still preliminary benefits. In contrast, DPP-4i demonstrate mixed results, and thiazolidinediones remain theoretically interesting but clinically impractical.

## 5. Conclusions

Glycemic management is central to COVID-19 care, with patients’ metabolic profiles influencing risk of respiratory decline and severe outcomes. Diabetes consistently elevates the risk of pneumonia, respiratory failure, and mortality, while SARS-CoV-2 infection can trigger new or persistent metabolic disturbances. These bidirectional interactions underscore the importance of targeted interventions and ongoing mechanistic research to improve patient outcomes.

## Figures and Tables

**Table 1:** Overview of Included Studies on Diabetes Mellitus and COVID-19. Publications are grouped by study design (clinical studies, case series, systematic reviews and meta-analyses, and narrative or mechanistic reviews) and ordered chronologically within each group. T2DM: type 2 diabetes mellitus; SGLT2: Sodium-glucose cotransporter 2; GLP-1RA: Glucagon-like peptide-1 receptor agonists; DPP-4: dipeptidyl peptidase-4, DKA: diabetic ketoacidosis.

*Clinical Studies*							
**Author (Year)**	**Country**	**Study Design**	**Cohort**	**Diabetes Type**	**Sample Size/Scope**	**Outcomes Evaluated**	**Main Conclusions**
Chen et al. (2020) [9]	China	Retrospective cohort	Hospitalized COVID-19 patients with diabetes; mean age 56 years; sex: 53.43% female/46.57% male; mortality 10.18%	Mostly T2DM	904 patients	Severity, mortality, glucose-lowering therapy	Diabetes and poor glycemic control associated with worse COVID-19 outcomes
Cariou et al. (2020) [10]	France	Multicenter observational cohort (CORONADO)	Adults with diabetes hospitalized with COVID-19; mean age 69.8 years; sex: 35.1% female/64.9% male; mortality 10.6%;	Predominantly T2DM (88.5%)	1317 patients	ICU admission, mortality	Diabetes associated with higher risk of severe COVID-19
Solerte et al. (2020) [11]	Italy	Multicenter retrospective case–control	Hospitalized COVID-19 patients, mean age 69 years; sex: 27% female/73% male;	Predominantly T2DM (95%);	338 patients	Mortality	Sitagliptin therapy associated with reduced mortality (18% compared to 37% of deceased patients) and greater number of hospital discharge (120 vs. 89).
Dalan et al. (2021) [12]	Singapore	Observational cohort	Patients with diabetes and COVID-19	Mostly T2DM	717 patients	Disease severity, immune markers	Antidiabetic therapies associated with different immune responses
Lalau et al. (2021) [13]	France	Observational cohort	Hospitalized COVID-19 patients with diabetes; 61.1% metformin users; sex: 36% female/64% male;	T2DM	2449 patients	Mortality	Metformin use associated with reduced mortality (8.2% vs. 16.1% in non-users at death at day 7; 16% vs. 28.6% vs. non-users in death at day 28)
Strollo et al. (2021) [14]	Italy	Observational study	Diabetes prevalence among COVID-19 deaths	T2DM	3711 patients	Mortality	No correlation between DPP-4 inhibitor use and mortality prevalence
Ohkuma et al. (2023) [15]	Japan	Retrospective cohort	Adults with pre-existing diabetes receiving routine care before and during the pandemic	Predominantly T2DM	3465 patients	Glycemic control	Pandemic conditions worsened metabolic control
Monda et al. (2023) [16]	Italy	Retrospective observational cohort	T2DM patients treated with GLP-1RA or SGLT2 inhibitors and hospitalized for COVID-19	T2DM	1916 patients	ICU admission, mortality	GLP-1RA and SGLT2 inhibitors associated with improved outcomes
Parlițeanu et al. (2025) [17]	Romania	Observational cohort	Hospitalized COVID-19 patients with pulmonary impairment; mean age 67.25; sex: 44.78% female/55.22% men, mortality associated with T2DM or altered glucose levels was 27.43%	Pre-existing DM and new-onset metabolic dysfunction	813 patients	Glucose metabolism	SARS-CoV-2 infection alters glucose homeostasis
*Case Series*							
**Author (Year)**	**Country**	**Study Design**	**Cohort**	**Diabetes Type**	**Sample Size/Scope**	**Outcomes Evaluated**	**Main Conclusions**
Suwanwongse & Shabarek (2021) [18]	USA	Case series	COVID-19 patients with DKA, age 18–64 years, sex: female 1 case (33%)/male 2 cases (67%)	New-onset diabetes	3 cases	DKA occurrence	COVID-19 may precipitate new-onset diabetes and DKA
*Systematic Reviews and Meta-analysis*							
**Author (Year)**	**Study Design**	**Cohort**	**Diabetes Type**	**Sample Size/Scope**	**Outcomes Evaluated**	**Main Conclusions**	
Shrestha et al. (2021) [19]	Systematic review and meta-analysis	COVID-19 patients with new-onset diabetes	New-onset diabetes	8 studies	Clinical outcomes	COVID-19 associated with increased incidence of newly diagnosed diabetes (Diabetes prevalence 19.7%; hyperglycemia 25.2%; mortality highest in new-onset diabetes (24.96%))	
Ganesh & Randall (2022) [20]	Systematic review and meta-analysis	COVID-19 patients with diabetes treated with metformin	Diabetes mellitus	32 studies	Mortality	Metformin therapy associated with reduced mortality	
Chen et al. (2022) [21]	Bayesian network meta-analysis	Diabetic COVID-19 patients receiving antidiabetic drugs	T2DM	42 studies	Clinical outcomes	Use of SLGT2 inhibitors linked to lower mortality	
Zhou et al. (2024) [22]	Systematic review and meta-analysis	Adults with COVID-19	New-onset diabetes	18 studies	Incident diabetes	SARS-CoV-2 infection associated with increased diabetes risk	
*Narrative and Mechanistic Reviews*							
**Author (Year)**	**Study Design**	**Topic**	**Diabetes Type**	**Sample Size/Scope**		**Main Conclusions**	
Drucker (2020) [23]	Narrative review	Diabetes and coronavirus pathophysiology	TD2M	Narrative review		ACE2 and DPP-4 pathways provide biological rationale for increased disease severity in diabetes.	
Pugliese et al. (2020) [24]	Narrative review	Diabetes as risk factor	Predominantly T2DM	Narrative review		Diabetes increases risk of severe COVID-19	
Chee et al. (2020) [25]	Narrative review	Interaction between diabetes and SARS-CoV-2	Diabetes mellitus	Narrative review		Delayed viral clearance observed in diabetic patients.	
Peric & Stulnig (2020) [26]	Narrative review	Diabetes management	Diabetes mellitus	Narrative review		Pandemic disrupted diabetes care	
Bassendine et al. (2020) [27]	Mechanistic review	DPP-4 signaling	Diabetes mellitus	Narrative review		DPP-4 may influence viral pathogenesis	
Scheen et al. (2020) [28]	Narrative review	Prognostic factors in diabetes and COVID-19	Diabetes mellitus	Narrative review		Diabetes and metabolic comorbidities associated with worse outcomes	
Landstra & de Koning (2021) [29]	Narrative review	Diabetes and COVID-19 risk	Diabetes mellitus	Review of major epidemiological studies		Population studies showed higher risk of severe COVID-19 in diabetes. Reported OR for mortality T1DM 3.51 and T2DM 2.03 compared to non-diabetes.	
Khunti et al. (2021) [30]	Narrative review	Hyperglycemia and COVID-19	Hyperglycemia/diabetes	Narrative review		COVID-19 linked to new-onset diabetes	
Raveendran & Misra (2021) [31]	Narrative review	Long-COVID	Diabetes mellitus	Narrative review		Post-COVID metabolic complications possible	
Wiernsperger et al. (2022) [32]	Mechanistic review	Metformin mechanisms	T2DM	Narrative review		Metformin may provide anti-inflammatory effects	
Gęca et al. (2022) [33]	Narrative review	Diabetes pathophysiology	Diabetes mellitus	Narrative review		Multiple metabolic pathways contribute to severe disease	
Dallavalasa et al. (2023) [34]	Narrative review	Diabetes and COVID-19 mechanisms	Diabetes mellitus	Narrative review		Pathophysiology and management considerations	
Foresta et al. (2023) [35]	Narrative review	Antidiabetic therapies and COVID-19 outcomes	T2DM	Narrative review		GLP-1 receptor agonists associated with improved outcomes	
Sakata (2024) [36]	Mechanistic review	Metformin molecular targets	T2DM	Narrative review		Anti-inflammatory signaling pathways	
Wang et al. (2024) [37]	Observational research review	Long-term COVID effects	Diabetes mellitus	Narrative review		Long-term metabolic consequences	
Rykova et al. (2025) [38]	Mechanistic review	SGLT2 inhibitors	T2DM	Narrative review		SLGT2 inhibitors reduce macrophage-driven inflammation	

**Table 2:** Overview of pharmacologic mechanisms of antidiabetic drugs.

Drug Class	Molecular/Cellular Target	Key Immunometabolic Effects
Metformin	AMPK activation, mitochondrial complex I	Anti-inflammatory, endothelial protection
SGLT2 inhibitors	SGLT2 blockade	Macrophage polarization, oxidative stress reduction, cardiorenal and endothelial protection
GLP-1 receptor agonists	GLP-1 receptor activation	Anti-inflammatory signaling, modulation of innate immunity
DPP-4 inhibitors	DPP-4/CD26	Immune signaling modulation
Thiazolidinediones (pioglitazone)	PPAR-γ activation, theoretical ACE2 modulation	Anti-inflammatory and metabolic regulation

## Data Availability

No new data were created or analyzed in this study. Data sharing is not applicable to this article.

## Supplementary Materials

The following supporting information can be downloaded at: https://www.mdpi.com/article/10.3390/ijms27083537/s1. Reference [64] is cited in Supplementary Materials file.

## Abbreviations

The following abbreviations are used in this manuscript:

ACE-2	angiotensin-converting enzyme-2
AMPK	adenosine monophosphate-protein kinase
COVID-19	coronavirus disease-19
DKA	diabetic ketoacidosis
DPP-4	dipeptidyl peptidase-4
DPP-4i	dipeptidyl peptidase-4 inhibitors
GLP-1RA	Glucagon-like peptide-1 receptor agonists
ICU	intensive care unit
IL-6	Interleukin-6
JBI	Joanna Briggs Institute
NOS	Newcastle-Ottawa Scale
SARS-CoV-2	severe acute respiratory syndrome coronavirus-2
SGLT-2i	Sodium-glucose cotransporter 2 inhibitors
T1DM	type 1 diabetes mellitus
T2DM	type 2 diabetes mellitus
TNF-α	Tumor necrosis factor-α
