# Supplementary material for: Diabetes Mellitus and COVID-19 in Adults: A Systematic Review of Pathophysiological Connections, Clinical Outcomes, and Therapeutic Considerations
Source: Int J Mol Sci. 2026 Apr 15;27(8):3537. doi: 10.3390/ijms27083537 (PMC13116372; doi:10.3390/ijms27083537)
Supplement: Supplementary file 1 [file ijms-27-03537-s001.zip › ijms-4181194-supplementary.pdf]

PRISMA 2020 Checklist

Section and Topic	Item #	Checklist item	Location where item is reported
**TITLE**			
Title	1	Identify the report as a systematic review.	Title ("Diabetes Mellitus and COVID-19 in Adults: **A Systematic Review** of…)"
**ABSTRACT**			
Abstract	2	See the PRISMA 2020 for Abstracts checklist.	Abstract (structured summary including objectives, eligibility criteria, databases, and results)
**INTRODUCTION**			
Rationale	3	Describe the rationale for the review in the context of existing knowledge.	Introduction, paragraph starting Introduction "Diabetes mellitus appeared to be the most common comorbidity…"
Objectives	4	Provide an explicit statement of the objective(s) or question(s) the review addresses.	Introduction, paragraph starting "This review aims to analyze research published…"
**METHODS**			
Eligibility criteria	5	Specify the inclusion and exclusion criteria for the review and how studies were grouped for the syntheses.	Methods, paragraph starting "The meeting criteria for inclusions were…" and paragraph starting "The exclusion criteria were…"
Information sources	6	Specify all databases, registers, websites, organisations, reference lists and other sources searched or consulted to identify studies. Specify the date when each source was last searched or consulted.	Methods, paragraph starting "The literature search was performed using PubMed…"
Search strategy	7	Present the full search strategies for all databases, registers and websites, including any filters and limits used.	Methods, paragraph starting "The following words were used in combination…"
Selection process	8	Specify the methods used to decide whether a study met the inclusion criteria of the review, including how many reviewers screened each record and each report retrieved, whether they worked independently, and if applicable, details of automation tools used in the process.	Methods, paragraph starting "Study selection was made based on titles and abstracts…"
Data collection process	9	Specify the methods used to collect data from reports, including how many reviewers collected data from each report, whether they worked independently, any processes for obtaining or confirming data from study investigators, and if applicable, details of automation tools used in the process.	Methods, paragraph starting "Data extraction included: authors, year of publication…"
Data items	10a	List and define all outcomes for which data were sought. Specify whether all results that were compatible with each outcome domain in each study were sought (e.g. for all measures, time points, analyses), and if not, the methods used to decide which results to collect.	Methods, paragraph starting "Data extraction included…"; variables: "authors, year of publication, study design…"
	10b	List and define all other variables for which data were sought (e.g. participant and intervention characteristics, funding sources). Describe any assumptions made about any missing or unclear information.	Methods, paragraph starting "Data extraction included…"; variables: "authors, year of publication, study design…"
Study risk of bias assessment	11	Specify the methods used to assess risk of bias in the included studies, including details of the tool(s) used, how many reviewers assessed each study and whether they worked independently, and if applicable, details of automation tools used in the process.	Methods, paragraph starting "Risk-of-bias assessment was conducted using validated tools…"
Effect measures	12	Specify for each outcome the effect measure(s) (e.g. risk ratio, mean difference) used in the synthesis or presentation of results.	Not applicable (narrative synthesis; no quantitative effect measures reported)
Synthesis methods	13a	Describe the processes used to decide which studies were eligible for each synthesis (e.g. tabulating the study intervention characteristics and comparing against the planned groups for each synthesis (item #5)).	Materials and Methods, paragraph starting "Studies were grouped for synthesis

Section and Topic	Item #	Checklist item	Location where item is reported
			according to study design and thematic domains…”
	13b	Describe any methods required to prepare the data for presentation or synthesis, such as handling of missing summary statistics, or data conversions.	Not applicable
	13c	Describe any methods used to tabulate or visually display results of individual studies and syntheses.	Materials and Methods, paragraph starting “Study characteristics and key findings were summarized in structured tables…”
	13d	Describe any methods used to synthesize results and provide a rationale for the choice(s). If meta-analysis was performed, describe the model(s), method(s) to identify the presence and extent of statistical heterogeneity, and software package(s) used.	Materials and Methods, paragraph starting “Due to heterogeneity in study designs, populations, and reported outcomes…”
	13e	Describe any methods used to explore possible causes of heterogeneity among study results (e.g. subgroup analysis, meta-regression).	Not reported
	13f	Describe any sensitivity analyses conducted to assess robustness of the synthesized results.	Not applicable
Reporting bias assessment	14	Describe any methods used to assess risk of bias due to missing results in a synthesis (arising from reporting biases).	Not reported
Certainty assessment	15	Describe any methods used to assess certainty (or confidence) in the body of evidence for an outcome.	Not reported
**RESULTS**			
Study selection	16a	Describe the results of the search and selection process, from the number of records identified in the search to the number of studies included in the review, ideally using a flow diagram.	Materials and Methods, paragraph starting “The initial search produced 412 records…”; Figure 1
	16b	Cite studies that might appear to meet the inclusion criteria, but which were excluded, and explain why they were excluded.	Not explicitly reported in Results
Study characteristics	17	Cite each included study and present its characteristics.	Results, paragraph starting “As detailed in Table 1, the body of evidence included 30 studies…”; Table 1
Risk of bias in studies	18	Present assessments of risk of bias for each included study.	Results, paragraph starting “The methodological quality of the included studies varied…”
Results of individual studies	19	For all outcomes, present, for each study: (a) summary statistics for each group (where appropriate) and (b) an effect estimate and its precision (e.g. confidence/credible interval), ideally using structured tables or plots.	Results,Table 1; sections 3.1–3.10, e.g., paragraph starting “Across multiple cohorts, diabetes was consistently associated…”
Results of syntheses	20a	For each synthesis, briefly summarise the characteristics and risk of bias among contributing studies.	Results, paragraph starting “Across included studies…” and paragraph starting “The methodological quality of the included studies varied…”
	20b	Present results of all statistical syntheses conducted. If meta-analysis was done, present for each the summary estimate and its precision (e.g. confidence/credible interval) and measures of statistical heterogeneity. If comparing groups, describe the direction of the effect.	Not applicable (no meta-analysis performed)
	20c	Present results of all investigations of possible causes of heterogeneity among study results.	Not applicable (no analysis of heterogeneity performed)

Section and Topic	Item #	Checklist item	Location where item is reported
	20d	Present results of all sensitivity analyses conducted to assess the robustness of the synthesized results.	Not applicable (no sensitivity analyses performed)
Reporting biases	21	Present assessments of risk of bias due to missing results (arising from reporting biases) for each synthesis assessed.	Not reported (no assessment of reporting bias performed)
Certainty of evidence	22	Present assessments of certainty (or confidence) in the body of evidence for each outcome assessed.	Not reported (no assessment of certainty of evidence performed)
**DISCUSSION**			
Discussion	23a	Provide a general interpretation of the results in the context of other evidence.	Discussion, paragraph starting “A unifying theme across the studies reviewed is the impairment of the immune response…”
	23b	Discuss any limitations of the evidence included in the review.	Discussion, paragraph starting “The evidence included in this review has several limitations…”
	23c	Discuss any limitations of the review processes used.	Discussion, paragraph starting “This review also has limitations related to the review process…”
	23d	Discuss implications of the results for practice, policy, and future research.	Conclusions, paragraph starting “Glycemic management is central to COVID-19 care…”
**OTHER INFORMATION**			
Registration and protocol	24a	Provide registration information for the review, including register name and registration number, or state that the review was not registered.	Data Availability Statement, sentence starting “This systematic review was registered in the OSF Registries…”
	24b	Indicate where the review protocol can be accessed, or state that a protocol was not prepared.	Data Availability Statement, sentence containing “https://osf.io/7pm53”
	24c	Describe and explain any amendments to information provided at registration or in the protocol.	Not reported
Support	25	Describe sources of financial or non-financial support for the review, and the role of the funders or sponsors in the review.	Funding, sentence starting “The Article Processing Charge was funded…”
Competing interests	26	Declare any competing interests of review authors.	Conflicts of Interest, sentence stating “The authors declare no conflicts of interest.”
Availability of data, code and other materials	27	Report which of the following are publicly available and where they can be found: template data collection forms; data extracted from included studies; data used for all analyses; analytic code; any other materials used in the review.	Data Availability Statement, sentence starting “This systematic review was registered in the OSF Registries…”

From: Page MJ, McKenzie JE, Bossuyt PM, Boutron I, Hoffmann TC, Mulrow CD, et al. The PRISMA 2020 statement: an updated guideline for reporting systematic reviews. BMJ 2021;372:n71. doi: 10.1136/bmj.n71
